# Monitoring suPAR levels in post-kidney transplant focal segmental glomerulosclerosis treated with therapeutic plasma exchange and rituximab

**DOI:** 10.1186/s12882-018-1177-x

**Published:** 2018-12-17

**Authors:** Nada Alachkar, Jing Li, Dany Matar, Vikas Vujjini, Sami Alasfar, Melissa Tracy, Jochen Reiser, Changli Wei

**Affiliations:** 1 Department of Medicine, Division of Nephrology, The Johns Hopkins University School of Medicine, Baltimore, USA; 2 Department of Medicine, Rush University Medical Center, 1735 W Harrison ST, Cohn Bldg, 7th Floor, Suite 716, Chicago, IL 60612 USA; 3 Department of Medicine, Sinai Hospital, Baltimore, USA; 4 Division of Nephrology, Johns Hopkins Hospital, 600 Wolfe St. Carnegie 344B, Baltimore, MD 21287 USA

**Keywords:** Kidney transplant, FSGS, suPAR, Therapeutic plasma exchange

## Abstract

**Background:**

Therapeutic plasma exchange (TPE) is an important therapy for recurrent focal segmental glomerulosclerosis (rFSGS) post kidney transplant. suPAR has been causally implicated in rFSGS, and shown to be a unique biomarker for the occurrence and progression of chronic kidney disease. This study was targeted to evaluate the application of monitoring suPAR in TPE treated rFSGS.

**Methods:**

A retrospective (*n* = 19) and a prospective (*n* = 15) cohort of post transplant FSGS patients treated with TPE and rituximab were enrolled. We measured serum suPAR levels before and after the combined therapies, and assessed the role of suPAR changes on proteinuria reduction and podocyte β3- integrin activity.

**Results:**

Treatment with TPE and rituximab resulted in significant decrease in proteinuria and suPAR levels. Among the variables including baseline suPAR, serum creatinine, proteinuria, eGFR, age at diagnosis, age at transplantation, transplantation numbers, time to recurrence, and TPE course numbers, only the reduction in suPAR levels and baseline proteinuria significantly correlated with the changes in proteinuria after treatment, with the former performed better in predicting proteinuria alteration. Additionally, the mean podocyte β3 integrin activity significantly decreased after TPE and rituximab treatment (1.10 ± 0.08) as compared to before treatment (1.34 ± 0.08), *p* < 0.05. Only the reduction in suPAR predicted the response to therapies with an odds ratio of 1.43, 95% CI (1.02, 2.00), *p* < 0.05.

**Conclusions:**

Serum suPAR levels reduced significantly after TPE and rituximab treatment in post transplant FSGS patients. The reduction in suPAR levels may be utilized to assess the changes in proteinuria and monitor the response to the therapies. Larger, multi-centered prospective studies monitoring serum suPAR levels in TPE managed post transplant FSGS are warranted.

## Background

Focal segmental glomerulosclerosis (FSGS) is a progressive renal disease with high probability for recurrence after renal transplantation [1, 2]. Therapeutic plasma exchange (TPE) is an extracorporeal blood purification technique designed for the removal of large molecular weight substances from the plasma. Since the first report in 1985 [3], TPE has become an important therapy with variable success in recurrent FSGS (rFSGS) [4, 5]. The application of TPE was originated from the concept of the disease causative circulating factor(s) in rFSGS [6].

Insights into podocyte biology have identified plasminogen activator, urokinase receptor (uPAR) as an important component in the maintenance of a functioning podocyte foot process structure that is regulated by lipid-dependent activation of αvβ3 integrin [7]. uPAR is a glycosyl-phosphatidylinisotol (GPI)-anchored protein, which can be released from the plasma membrane as a soluble molecule (suPAR) by cleavage of the GPI anchor or secreted directly from cells as an alternative transcript [8]. We have identified suPAR as a circulating factor implicated in the majority of FSGS cases [9, 10]. Yet, the relevance of suPAR to kidney injury has been surprisingly broadened recently. Several longitudinal studies in a variety of patient cohorts (cardiac risk, healthy middle-aged, pre-diabetic, dialysis patients) have found that baseline circulating suPAR levels predict chronic kidney disease (CKD) incidence and progression [11–13], strongly suggesting the application of circulating suPAR as a biomarker for monitoring CKD.

As TPE has been found to remove suPAR from blood circulation in several studies [9, 14–16], we sought to evaluate the application of serum suPAR as a biomarker monitoring TPE treated rFSGS patients in this study.

## Methods

### Study cohorts

We conducted both retrospective and prospective studies of patients with FSGS as the cause of end stage renal disease (ESRD). Only patients who received TPE alone or together with rituximab for recurrent FSGS treatment were enrolled. All subjects received immunosuppressive therapy consisted of induction with thymoglobulin (1.5 mg/kg/day) and steroid (500 mg/day), followed by maintenance treatment with tacrolimus, mycophenolate mofetil and prednisone. Both studies were approved by the institutional review board (IRB) of Johns Hopkins Hospital. In the retrospective study cohort, we included 19 adult renal transplant recipients who underwent renal transplantation between September 1, 2008 to December 31, 2011 in our center and developed rFSGS after kidney transplantation. For the purpose of our prospective study, we enrolled 15 patients who were transplanted in our center between August 1, 2011 and May 31, 2015 and developed rFSGS post transplant. We followed these participants as per our research protocol and collected serum, and plasma waste samples before and after each TPE. We also evaluated the subjects’ response to TPE treatment. Response to therapy is defined as reduction of proteinuria to less than 1 g/g, or the reduction of proteinuria by more than 50%. The demographic and clinical characteristics of both the retrospective and the prospective cohorts are shown in Table 1.

**Table 1:** Demographic and Clinical Characteristics of Participants

	Retrospective (*n* = 19)	Prospective (*n* = 15)	*P* value
Male, n (%)	8 (42)	10 (67)	0.185
Black, n (%)	11 (58)	5 (33)	0.185
Mean Age at Tx, yr. ± SD	40 ± 12	38 ± 16	0.703
Mean Age at native FSGS Diagnosis, yr. ± SD	29 ± 8	30 ± 17	0.859
Median Duration on Dialysis, yr. (IQR)	2.5 (1.5, 8.0)	3.8 (1.3, 6.0)	0.888
Pre-Tx Urine, n (%)	13 (68)	6 (40)	0.165
Mean Pre-Transplant Proteinuria, g/g ± SD	9.43 ± 12	8.88 ± 7.32	0.673
Primary Pre-Tx Diagnosis, n (%)			
FSGS	17 (89)	15 (100)	0.492
Other*	2 (11)		
No. of Transplants, n (%)			
1	12 (63)	8 (53)	0.659
2	4 (21)	5 (33)	
3	3 (16)	1 (7)	
Living Donor, n (%)	12 (63)	11 (73)	0.469
Related	5 (26)	3 (20)	
Unrelated	7 (37)	8 (53)	
ABO-Incompatible Tx, n (%)	4 (21)	1 (7)	0.633
Median Time to Post-Tx FSGS, days (IQR)	31 (5, 238)	33 (5, 299)	0.589
Mean Proteinuria at time of Post-Tx FSGS, g/g ± SD	4.7 ± 3.6	3.2 ± 2.8	0.366
Mean Peak Proteinuria, g/g ± SD	10.94 ± 11.61	4.6 ± 3.25	0.061
Median Serum Creatinine at time of Post-Tx FSGS, mg/dL (IQR)	2.5 (1.7, 3.5)	1.5 (1.3, 2.4)	0.310
Median eGFR at time of Post-Txp FSGS, mg/dL (IQR)	32 (14, 44)	55 (29, 61)	0.943
Median TPE (IQR)	14 (10, 27)	10 (10, 19)	0.447

*yr* year, *SD* standard deviation, *IQR* interquartile range, *g* gram, *Tx* transplant

The diagnosis of recurrent and de novo FSGS was made by the new onset of proteinuria as measured by urine protein-creatinine ratio (UPCR) of more than 1 g/g and confirmed by kidney biopsy. Kidney biopsy in early stages showed only significant podocyte effacement on electron microscopy. Classical findings of light microscopic changes are also seen.

### suPAR measurement

The measurement of serum suPAR was performed using a Human uPAR Quantikine ELISA kit (R&D Systems Inc) following the manufacturer’s instruction [9, 10]. Standards were run three times to calculate the intra-assay coefficient of variation (CV). The mean and SD for standard 1, standard 2, and so forth were used to derive the CV before averaging the CV of each standard. The inter-assay CV was derived by calculating the mean and SD for standard 1 (e.g.*,* measurement day 1 and day 2), standard 2 (day 1 and day 2), and so forth to derive the CV and then average the CV. Both the intra-assay and inter-assay CVs were < 5% for suPAR.

### Induced podocyte β3 integrin activity assay

To semi-quantitatively examine the effect of FSGS patient sera on podocyte β3 integrin activity, a human podocyte cell line was cultured at 37 °C for 14 days for complete differentiation [17]. The cells were then incubated in 5% of FSGS patient serum for 24 h with lipopolysaccharide (LPS) as a positive control. Next, the cells were fixed with 4% paraformaldehyde (PFA) and processed for immunofluorescence staining for AP5 (Blood Center of Wisconsin) and paxillin (Millipore). AP5 is an antibody detecting the active state of β3 integrin by recognizing the unfolding N-terminal epitope GPNICT upon the activation of the integrin [18]. After immunostaining, confocal (Leica) images were taken to quantify the AP5 and paxillin intensity for each sample treatment. Paxillin signal was used to correct AP5 signal for each treatment. The relative AP5 signal (AP5/paxillin ratio) from each patient serum was then normalized against that of normal blood donor included in each assay for final report [15]. To control for suPAR specificity, the cells were co-incubated with both FSGS sera and suPAR blocking antibody. The normalized AP5 value from normal serum treated podocytes was 1. The relative AP5 value of 1.05 or more obtained from patient serum treated podocytes was considered abnormal.

### Statistical analysis

For continuous variables, data are expressed as mean ± SEM or median with interquartile range as appropriate. Categorical variables were expressed as percentages. The demographic and clinical characteristics of patient and control participants were compared using the *t* test, or the Fisher’s exact test for categorical variables. Multiple linear or logistic regression analyses were performed to evaluate the association between serum suPAR and the variables of interest while controlling for age, sex, and other potential confounders with SPSS software. The relative change in suPAR after TPE treatment was calculated as per 10% reduction from before TPE treatment. The relative change of proteinuria in terms of UPCR was calculated as 100 x (UPCR before treatment-UPCR after treatment)/UPCR before treatment. All statistical tests were two tailed. *P* values < 0.05 were considered significant.

## Results

### Single course of TPE on suPAR removal

**Fig. 1:**
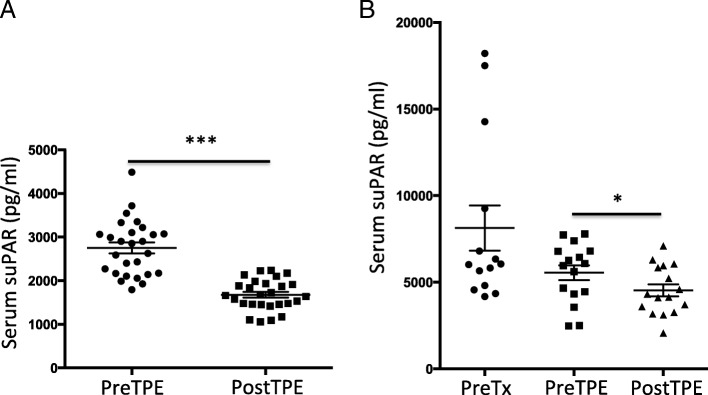
TPE reduced serum suPAR levels **a** Single course of TPE significantly reduced serum suPAR levels. ***, *p* < 0.001. **b** TPE alone or combined therapy decreased serum suPAR levels in retrospective cohort of post transplant FSGS. * *p* < 0.05

To look at the immediate effect of TPE on serum suPAR levels, we compared serum suPAR right before and after a single course of TPE. We found that single course of TPE could remove on average 37% of serum suPAR (Fig. 1a). Simultaneously, suPAR was detected in the pheresis waste bags, ranging from 1149 pg/ml to 2417 pg/nl with an average suPAR value of 1848 pg/ml. This is in consistent with previous reports [9, 14–16], and indicates that TPE could effectively decrease serum suPAR levels by removing suPAR from the blood circulation.

### Historical cohort

**Fig. 2:**
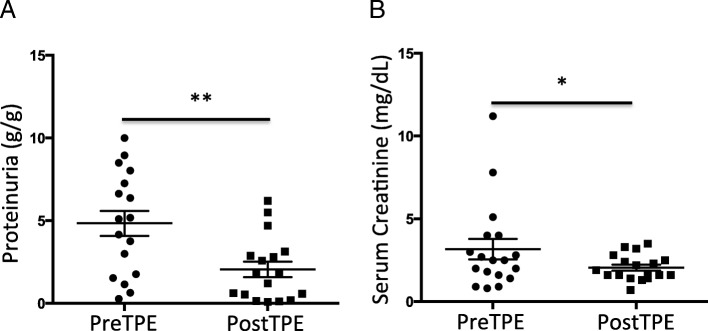
Effects of TPE alone or combined therapy in retrospective cohort **a**. Proteinuria was significantly decreased after TPE therapy. ** *p* < 0.01. **b** Serum creatinine was reduced after TPE therapy. * *p* < 0.05

We performed a retrospective study, in which we analyzed 19 post-transplant (post-Tx) FSGS patients, 17 of them had rFSGS, and 2 had de novo FSGS (Table 1), 42% of them were male. The mean ± SD age at transplant was 40 ± 12 years old. Median time to post-Tx FSGS (IQR) was 31 (5, 238) days. All patients were treated with TPE, and 12 received rituximab as well. Serum was collected before transplantation, after transplantation at the time of post-Tx FSGS diagnosis, and before and after TPE sessions for suPAR measurement. Each patient received a median (IQR) of TPE 14 (7, 82) sessions (Table 1). As shown in Fig. 1b, mean serum suPAR was higher before transplantation comparing to that at the time of post-Tx FSGS diagnosis (8131 ± 1300 pg/ml before Tx versus 5551 ± 429.2 pg/ml post-Tx, *p* = 0.056). One TPE session resulted in significant reduction in serum suPAR level (5551 ± 429 pg/ml before TPE vs 4532 ± 351 pg/ml post TPE, *p* < 0.05). By the end of treatment with TPE and rituximab, the mean proteinuria significantly decreased from 4.84 ± 0.76 g/g to 2.06 ± 0.47 (*p* < 0.01, Fig. 2a). The median serum creatinine levels were significantly decreased as well (2.5 mg/dL before therapies versus after 1.8 mg/dL after therapies, *p* < 0.05, Fig. 2b).

### Prospective cohort

**Fig. 3:**
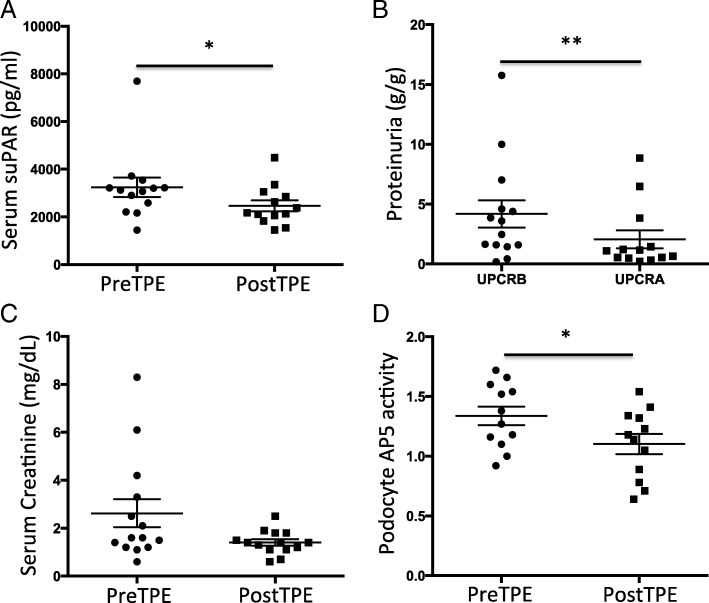
Effects of TPE combined therapy in prospective cohort **a**. Serum suPAR levels were significantly reduced after treatment. * *p* < 0.05. **b** Proteinuria was significantly decreased after treatment. ** *p* < 0.01. **c** Serum creatinine was marginally but not significantly improved after combined therapy. **d** The patient serum induced podocyte AP5 activity was significantly reduced after treatment. * *p* < 0.05

**Fig. 4:**
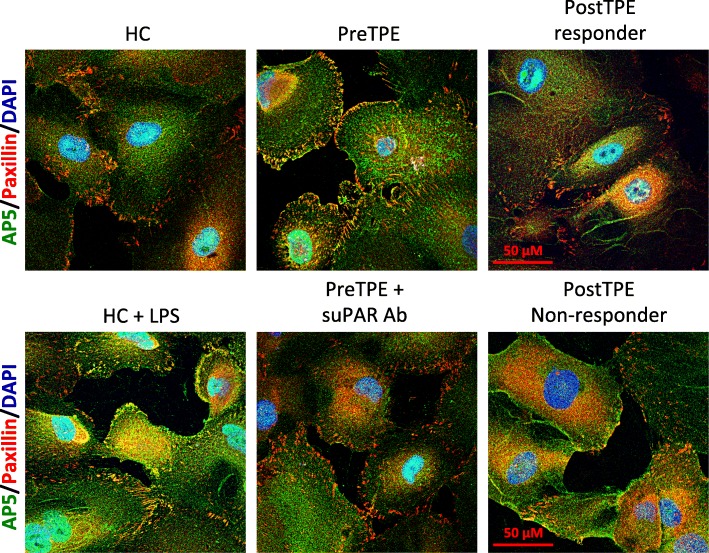
Immunostaining of podocyte AP5 activity To look at the effect of post transplant FSGS patient sera on cultured human podocyte αvβ3 integrin activity, podocytes were immunostained with AP5 antibody after respective overnight treatments. While sera from normal subject (HC) generated only minimum amount of AP5 signal, before TPE treatment (PreTPE) sera harvested from post transplant FSGS patients induced AP5 activity apparently in podocytes, which could be blocked by co-incubation with anti-human suPAR antibody. In contrast, the enhanced podocyte AP5 activity was abolished with the post-TPE treatment sera (PostTPE) collected from therapy responder but not with those post-TPE sera obtained from non-responder

To validate our findings of the retrospective study and assess the role of suPAR in monitoring the response to TPE in post-Tx FSGS, we performed a prospective study of 15 patients with rFSGS. As indicated in Table 1, 67% of them were male and mean ± SD age at transplantation was 43 ± 15 years old. The median time to rFSGS was 33 (5, 299) days. All these patients received TPE and rituximab. The median (IQR) TPE was 10 (10, 52) sessions. Serum was collected to measure serum suPAR and creatinine levels, and urine was collected for proteinuria assessment before and after TPE treatment. We found that one TPE resulted in significant reduction in serum suPAR levels to from 3240 ± 409.4 pg/ml, to 2486 ± 229 pg/ml (*p* < 0.05, Fig. 3a). Similarly, proteinuria decreased significantly as well (4.19 ± 1.14 g/g before vs 2.07 ± 0.75 g/g after treatment, *p* < 0.01, Fig. 3b). The median serum creatinine reduced but did not reach any statistical significance (Fig. 3c). Building on our previous findings that elevated circulating suPAR could induce podocyte β3 integrin activity and thus kidney injuries [9], we performed cultured human podocyte based β3 integrin activity assay by incubating podocytes with post-Tx FSGS patient sera harvested before and after TPE treatments (Fig. 4). The generated AP5 activity was corrected against that of normal sera from healthy subjects. Co-incubation of the patient sera with anti-human suPAR antibody was applied in order to control for the suPAR effect (Fig. 4). In consistent with the changes of serum suPAR levels and proteinuria, the mean β3 integrin activity in terms of AP5 intensity significantly decreased after treatment (1.10 ± 0.08) as compared to before treatment (1.34 ± 0.08, *p* < 0.05, Fig. 3d). Before TPE and rituximab combined therapies, 11 out of 12 patients had high podocyte AP5 value, while 7 of 12 patients had high AP5 after treatment. Out of 5 patients with AP5 normalized after treatment, only 1 (20%) did not respond to TPE and rituximab. Among the 7 patients with persistent high podocyte AP5 values after treatment, 5 (71.4%) did not respond to the combined therapies.

### Correlation of serum suPAR modification to clinical outcome

Next, we analyzed the distribution of both retrospective and prospective cohorts and found that the demographic and clinical characteristics are similar (Table 1), thus we combined the two cohorts together for further analysis. First, we examined the bivariate correlations between the continuous variables, including serum suPAR, serum creatinine, eGFR, and UPCR before TPE treatment; age at diagnosis, age at transplantation, transplant number, TPE course numbers, as well as, the reduction in serum suPAR (dsuPAR), the reduction in UPCR (dUPCR) after treatment (Table 2). Pre-TPE levels of serum suPAR were associated with serum creatinine (*r* = 0.37, *p* < 0.05). Pre-TPE UPCR was correlated with serum creatinine (*r* = 0.47, *p* < 0.01), and transplant number (*r* = 0.38, *p* < 0.05). The reduction in UPCR after treatment was correlated with Pre-TPE UPCR (*r* = 0.42, *p* < 0.05), and the reduction in suPAR (*r* = 0.48, *p* < 0.01).

**Table 2:**
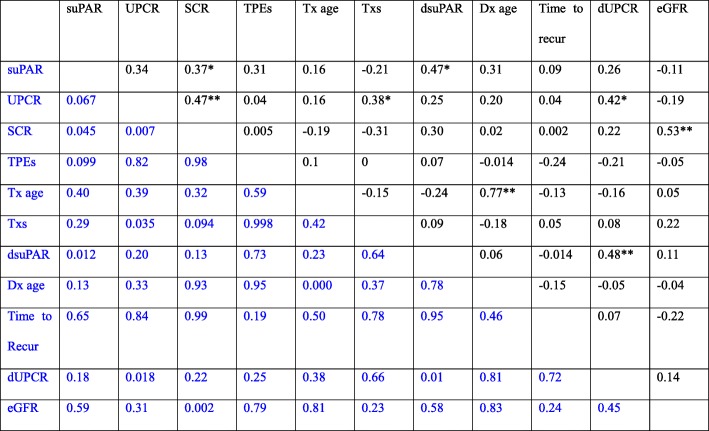
Correlations between different variables

Upper in black, Pearson *r* vlue; lower in blue, *p* value. dUPCR, the relative change in UPCR; dsuPAR, the relative change in serum suPAR levels; UPCR, before TPE urinary protein creatinine ratio; SCR, before TPE serum creatinine; Tx age, age at transplant; Tx#, transplant times; TPE#, the number of TPE courses treated; Dx age, age at diagnosis;

dUPCR = 100 x (UPCR PreTPE-UPCR PostTPE)/UPCR PreTPE;

dsuPAR = Per 10% reduction from PreTPE levels of suPAR

*Correlation is significant at the 0.05 level (2-tailed); ** Correlation is significant at the 0.01 level (2-tailed)

Then, we performed multiple linear regression analysis to evaluate the reduction in UPCR after treatment, controlling for Pre-TPE suPAR, UPCR, serum creatinine, eGFR, and patient gender, TPE number, age at transplant, transplant number, as well as the reduction in suPAR post-TPE treatment. We observed that the reduction in serum suPAR levels alone accounted for 23% proteinuria reduction. The reduction in serum suPAR levels and baseline UPCR together accounted for 35% proteinuria reduction after treatment, with the reduction in serum suPAR still predicting stronger than baseline UPCR (Table 3). Lastly, we performed a logistic multiple regression to look at the response to TPE and rituximab combined therapies, which was defined as UPCR < 1 g/g and/or the reduction in UPCR more than 50%, controlling for variables including the reduction in serum suPAR levels, Pre-TPE UPCR, eGFR, TPE number and age at transplant. We found that only the reduction in serum suPAR significantly predicts the response to the therapies with an odds ratio of 1.43, 95% CI (1.02, 2.00), *p* < 0.05.

**Table 3:** Multiple linear regression analysis of proteinuria change

Model		B	SEM	β	*P* value
1	Constant	20.84	13.22		
	Relative change in suPAR	9.84	3.54	0.48	0.01
2	Constant	−11.3	19.56		
	Relative change in suPAR	8.00	3.43	0.39	0.03
	Baseline UPCR	7.08	3.33	0.36	0.04

*UPCR* urine protein-creatinine ratio

## Discussion

Originating from the concept of circulating permeability factor(s), TPE has become an important therapy in recurrent FSGS patients. A recent systematic review of patients with rFSGS indicates that 71% of patients achieved full or partial remission after treatment with TPE [19]. Yet, there has been no biomarker available to gauge TPE therapy, largely due to the delay in identifying the responsible circulating factor(s). In this study containing both retrospective and prospective rFSGS cohorts, we found that serum suPAR was reduced after TPE treatments and the reduction in suPAR could predict the response to TPE and rituximab.

While suPAR has been considered to be an inflammatory marker, implicated in many medical conditions, its pathogenic involvement in kidney has been just unfolded. Several large and diverse cohorts have shown that baseline suPAR stands out as a unique biomarker in predicting the occurrence and progression of CKD, as well as the cardiovascular events with both adult and children patients [11–13]. In terms of FSGS, we have shown that suPAR as a circulating factor can contribute to the development of FSGS, via mechanisms that activate podocyte αvβ3 integrin [9]. Moreover, increased serum suPAR levels have been observed in a majority of primary FSGS patients and most rFSGS patients investigated [9, 10]. While not all follow-up studies have reached the same conclusions [20], and indeed further multicenter large cohort studies that could adopt the same study protocols are warranted, these findings highly suggest the implication of suPAR in FSGS patients.

Recently, we have shown that the degree of podocyte effacement correlated with suPAR levels at the time of rFSGS diagnosis, and that response to therapy resulted in significant reduction of suPAR level [21]. In this study, we found that circulating suPAR could be effectively reduced by a single course of TPE. This is in consistent with other reports [9, 14–16], indicating that suPAR can be managed by TPE. Yet, why serum suPAR bounced back significantly in some but not other studies [15], and how to control the rebound of serum suPAR and proteinuria deserve further investigation. Of the possible explanations for the discrepancy, the difference in clinical management protocols adopted at different institutes should be accountable at least partially.

In this study of the post-Tx FSGS patients, we found that TPE reduced serum suPAR levels and suPAR-induced podocyte αvβ3 integrin activity. More importantly, the decrease of serum suPAR is associated with the reduction of proteinuria. Controlling for variables including baseline serum creatinine, eGFR, proteinuria, age at transplantation, transplant numbers, and TPE course numbers, the reduction in suPAR stands out as the strongest predictor for proteinuria reduction after TPE treatments, account for 23% of proteinuria variance. In terms of response to therapy (UPCR< 1 g/g and/or UPCR reduction > 50%), out of many above analyzed variables, only the reduction in suPAR level can predict the outcome, indicating suPAR is an applicable biomarker in TPE managed post-Tx FSGS.

Our study has few limitations, in particular the small sample size of our cohort. Additionally, for our prospective study, we have only short-term outcome. Obviously, a multicenter prospective study that includes a larger cohort with long term follow up duration is warranted to address these issue. Nevertheless, monitoring and regulating suPAR levels in patients with rFSGS or other kidney disease deserve more attention, especially because experimental evidence is emerging that suggests inhibiting uPAR pathway by cyclo-RGDfv, uPAR antibody or most recently by UPARANT, a uPAR-derived small peptide with predominant anti-inflammatory action, could render renal protection [7, 9, 22].

## Conclusions

In this study of post transplant FSGS patients managed with TPE and rituximab, the reduction of suPAR after treatment contributed significantly to the reduction in proteinuria as well as to the response to therapy. These findings support monitoring of suPAR in TPE and rituximab managed recurrent and de novo FSGS.

## Abbreviations

CI: Confidence interval
CKD: Chronic kidney disease
CV: Coefficient of variation
eGFR: Estimated glomerular filtration ratio
ESRD: End stage renal disease
FSGS: Focal segmental glomerulosclerosis
GPI: Glycosyl-phosphatidylinisotol
IQR: Interquartile range
LPS: Lipopolysaccharide
PFA: Paraformaldehyde
SCR: Serum creatinine
SD: Standard deviation
SEM: Standard error of mean
suPAR: Soluble urokinase receptor
TPE: Therapeutic plasma exchange
Tx: Transplantation
UPCR: Urinary protein/creatinine ratio
